# Treating cancerous large airway stenosis with staging radioactive particle implantation guided by computed tomography and fiber bronchoscopy: a clinical study

**DOI:** 10.1186/s12957-017-1216-2

**Published:** 2017-08-03

**Authors:** Yu Mao, Xiuyan Yang, Ming Li, Wei Guo, Wenhua Zhang

**Affiliations:** Department of Thoracic Surgery, The First People’s Hospital of Hohhot, Hohhot, Inner Mongolia 010020 China

**Keywords:** Fiber bronchoscope, Lung cancer, Radioactive particle

## Abstract

**Background:**

The purpose of this study is to investigate the clinical effectiveness of staging radioactive particle implantation guided by computed tomography (CT) and fiber–optic bronchoscopy in treating cancerous large airway stenosis.

**Methods:**

A total of 102 patients were included; 57 had undergone staging radioactive particle implantation guided by CT and fiber bronchoscopy and 45 did not. Patients were evaluated by CT and fiber–optic bronchoscopy to determine the feasibility of the implantation of radioactive seeds for the treatment of cancerous large airway stenosis. The treatment planning system (TPS) was used to plan the doses. Radioactive seeds were implanted using fiber–optic bronchoscopy. One week later, CT-guided implantation of radioactive seeds was performed.

**Results:**

The clinical evaluation showed complete, partial, slight, and non-response in 38, 14, 5, and 0 patients, respectively. None of the patients were found with serious complications. The diameter of the affected airway, Karnofsky score, dyspnea index, survival, and quality of life of the patients in both groups was significantly higher and significantly different after the treatment (*P* < 0.05). The dyspnea index was significantly lower in the treatment group as compared with the control group (*P* < 0.001).

**Conclusion:**

CT- and fiber bronchoscopy-guided staging radioactive particle implantation has definite treatment effectiveness in treating cancerous large airway stenosis. It should be widely used in clinical practices.

## Background

Radioactive particles are being more and more commonly used to treat lung cancer in China. These particles are generally implanted percutaneously or by surgery [1, 2], but the implantation can be very difficult in some patients due to the location of the tumor, especially for cancerous large airway stenosis. In such patients, the radioactive particles could be implanted permanently under the guidance of fiber bronchoscopy. The radioactive particles kill the tumor cells continuously, and hence achieve the treatment effects that external irradiation could not achieve [3, 4]. Only a few studies have reported this method to date. Since the beginning of the twenty-first century, permanent radioactive particle implantation guided by fiber bronchoscopy has been conducted in European countries to treat cancerous large airway stenosis [1, 2]; nevertheless, anecdotal cases were treated using this method in China since 2007 and they achieved satisfactory treatment effectiveness. For tumors distant from the airway, CT-guided percutaneous radioactive particle implantation can be performed to ensure that the tumors are irradiated continuously from all directions [5–7].

From March 2011 to December 2015, 57 patients with cancerous large airway stenosis were successfully treated at our department using computed tomography (CT) and fiber bronchoscopy-guided staging radioactive particle implantation. The effectiveness of treatment in these patients was satisfactory. The treatments and outcomes were reported in this retrospective study.

## Methods

### Clinical characteristics

From March 2011 to December 2015, 102 patients with moderately to advanced non-small cell lung cancer (NSCLC) were selected for this retrospective study and randomly divided into two groups: the treatment and control groups. Clinical characteristics are presented in Table 1. Sex, age, and stages of the patients in both groups were all comparable.

**Table 1:** Characteristics of the patients

	Treatment (*n* = 57)	Controls (*n* = 45)	*P*
Sex			>0.05
Male	37	27	
Female	20	18	
Age			>0.05
Mean	64	64	
Range	52–75	47–79	
Implanted seeds by bronchoscopy			–
Total	399	–	
Mean	7	–	
Range	5–10	–	
Implanted seeds using CT			–
Total	399	–	
Mean	33	–	
Range	20–46	–	
Chemotherapy, *n* (%)	57 (100.0)	45 (100.0)	–
Serious complications, *n* (%)	0	0	–
Mild complications, *n* (%)		–	–
Slight hemoptysis	3 (5.3)		
Decreased WBC	4 (7.0)		
Pneumothorax, low fever, particle shifting, and mild pulmonary fibrosis	2 (3.5)		
Pneumothorax, bleeding, and mild adverse radiation reaction	1 (1.8)		

CT- and fiber bronchoscopy-guided staging radioactive particle implantation were performed to treat the cancerous large airway stenosis. The patients in both groups met the following criteria: (1) with cancerous large airway stenosis; (2) the lung cancer was confirmed by pathological or histological examinations; (3) could not receive radical operation due to various reasons (e.g., serious heart, liver, or kidney diseases; serious infection, bleeding, or tendency to bleed; contraindication to general anesthesia; or the patient had already been operated for lung cancer and further reoperation is impossible); and (4) without absolute contraindication to fiber bronchoscopy treatments. For the patients in the treatment group, sealed ^125^iodine (^125^I) radioactive sources with a surface activity of 0.7 mCi were implanted inside the airway lumen and outside the airway wall under the guidance of fiber bronchoscopy. After 1 week, sealed ^125^I radioactive sources with a surface activity of 0.7 mCi were percutaneously implanted under CT guidance. Chemotherapy was performed 8 months after the operation (chemotherapy strategy: vinorelbine + carboplatin). The patients in the control group had received chemotherapy only.

### Equipment

An Olympus fiber bronchoscope was used in this study. Sealed ^125^I sources were obtained from the HTA Co., Ltd. (Beijing, China). The particles were with titanium enclosure, 0.8 mm in diameter and 4.8 mm in length. The radioactivity of these cylindrical particles was 0.7 mCi, half-life was 59.6 days, and available irradiation range was 17 mm [8, 9].

### Operation procedures

The clinical presentations and CT images were analyzed comprehensively to assess the feasibility of using CT- and fiber bronchoscopy-guided staging radioactive particle implantation to treat cancerous large airway stenosis. Then, the anatomical relationships between the tumors and blood vessels inside the airway lumen, at the airway wall, and outside the airway lumen as well as the positions and number for the radioactive particle implantation were clarified using the treatment planning system (TPS). After single-lumen endotracheal tube (size 8) anesthesia, interventional implantation device was used to punctuate the lesions under the guidance of fiber bronchoscopy, and radioactive particles were implanted, with the distance between each particles of about 0.5–1 cm. For the tumors distant from the airway, re-examination with CT scanning was performed 1 week after the bronchoscopy-guided radioactive particle implantation. The TPS was used to clarify the number and positions of the radioactive particle implantation again, and CT scanning was used to guide the radioactive particle implantation. The patients were observed routinely for 48 h after the operation, and hemostatic and anti-inflammation treatments were used.

### Criteria for effectiveness evaluation

For the evaluation of treatment effectiveness, the overall quality of life, Chinese version of EORQLQ-LCl3, indicators of airway stenosis, and difference in survival were compared to comprehensively evaluate the changes in the quality of life from the physiological and psychological aspects. The dyspnea index was classified as follows: 0 indicates no dyspnea when climbing stairs, 1 indicates dyspnea when climbing stairs, 2 indicates dyspnea when walking, 3 indicates dyspnea when moving, and 4 indicates dyspnea when lying quietly in bed [10, 11].

### Statistical analysis

SPSS 13.0 was used for the statistical analysis. The data were presented using mean ± standard deviation and compared with the Student *t* test. *P* < 0.05 was considered as statistically significant.

## Results

### Clinical outcomes

No serious complication was found during this study. The complications observed in the course of the study period were as follows: slight hemoptysis (3/57, 5.26%); decreased white blood cell count (4/57, 7.01%); pneumothorax, low-grade fever, particle shifting, and mild pulmonary fibrosis (2/57, 3.51%); and pneumothorax, bleeding, and adverse radiotherapy reaction (1/57, 1.75%). All these complications were relatively mild and quickly recovered after symptomatic treatments.

**Fig. 1:**
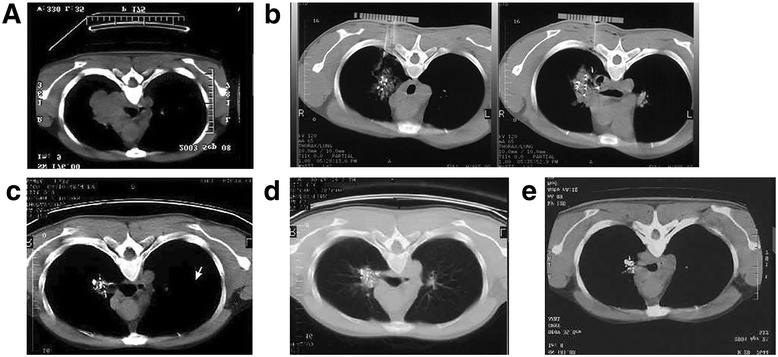
Contrast-enhanced computed tomography (CT) showing the lesion of one typical patient at different time points: pre-operation (**a**), intra-operation (**b**), and 1 (**c**), 3 (**d**), and 6 (**e**) months post-operation. The 125I particles gathered slowly after implantation and the lesion fully resolved 6 months after operation

Re-examinations with enhanced CT scanning after the operation were performed to determine the effectiveness of the treatment. The enhancement of the lesions decreased gradually at 1 week and 1, 3, 6, and 12 months after the operation, and the ^125^I particles gathered slowly (Fig. 1). The obstructed airways were restored obviously, and the obstructive pneumonia and pulmonary atelectasis were improved.

### Survival

The patients were followed up for 6–18 months. Only four patients died of systemic failure, whereas all the other patients survived. The mean survival time was 4.57 ± 2.05 months for the 45 patients in the control group and 11.13 ± 2.08 months for the 57 patients in the treatment group (*t* = 6.152, *P* < 0.05), suggesting that the survival time was significantly longer in the treatment group than that in the control group (Table 2).

**Table 2:** Comparisons of the survival between the treatment and control groups

Groups	Patients (*n*)	Survival time (months)
Treatment	57	11.13 ± 2.08*
Controls	45	4.57 ± 2.05

*P < 0.05 vs. controls

The results showed that the indicators of airway stenosis in the treatment group were decreased significantly after treatment. In addition, the rate of cross-section of airway stenosis was significantly different between both groups after treatment (*t* = 5.714, *P* < 0.05). The dyspnea index was also significantly different between both groups (*t* = 4.691, *P* < 0.05). These findings suggest that the indicators of airway stenosis were significantly better in the treatment group than that in the control group (Table 3).

**Table 3:** Comparisons of the airway stenosis indicators

Groups	Patients (*n*)	Rate of airway cross-section stenosis	Dyspnea index
Treatment	57		
Before treatment		6.86 ± 1.68	12.73 ± 2.14
After treatment		2.57 ± 4.43*	5.25 ± 3.44*
Controls	45		
Before treatment		5.57 ± 2.07	11.84 ± 2.65
After treatment		6.29 ± 4.43	15.71 ± 4.02

*P < 0.05 vs. controls after treatment

### Quality of life

The overall quality-of-life assessment and Chinese version of EORQLQ-LCl3 scale were used to evaluate the changes in the quality of life of the patients (Table 4). EORQLQ-LCl3 and overall quality of life before treatment were not significantly different between both groups (*t* = −0.273 and 1.139, respectively, *P* > 0.05), suggesting that the preoperative data were comparable between both groups. The paired *t* test showed that the EORQLQ-LCl3 and overall quality of life improved significantly in both groups after treatment (*t* = 3.591 and 25.257, respectively, *P* < 0.05). The independent *t* test showed that the EORQLQ-LCl3 and overall quality of life in the treatment group were significantly better than those in the control group after the treatment (*t* = −3.672, *P* < 0.05).

**Table 4:** Comparisons of the overall quality of life and the Chinese version of EORQLQ-LCl3

Groups	Patients (*n*)	EORQLQ-LCl3	Physiological functions	Clinicians and disease	Emotions and social and family situations	Overall quality of life
Treatment						
Before treatment	57	21.18 ± 2.63	15.41 ± 2.28	19.15 ± 2.39	24.13 ± 3.91	98.87 ± 5.37
After treatment	57	25.39 ± 3.76*	17.89 ± 3.01*	25.64 ± 3.12*	39.26 ± 3.42*	125.38 ± 6.37*
Controls						
Before treatment	45	20.67 ± 2.97	15.68 ± 2.37	19.86 ± 2.54	23.42 ± 3.87	98.18 ± 6.89
After treatment	45	14.32 ± 3.96	11.29 ± 2.57	13.41 ± 2.32	20.12 ± 2.87	87.13 ± 4.52

*P < 0.05 vs. before treatment

## Discussion

Advanced central type lung cancer could easily cause cancerous large airway stenosis, which severely affect the ventilation functions of the patients and reduce their quality of life; the evident dyspnea is the most common reason of non-cancer death of these patients [12–14]. Radioactive ^125^I particles are widely used for the treatment of various tumors. These particles could release low-power γ-rays continuously, with the available irradiation range of 17 mm and half-life of 59.6 days, continuously killing the tumor cells and achieve ideal treatment effectiveness. Minimally invasive treatment in the airway is the most effective methods for the treatment of cancerous large airway stenosis or obstruction, which could substantially improve the quality of life of the patients [15]. For tumors inside the airway lumen, the most commonly used methods include freezing, microwave, high-frequency electrotome, and laser therapies. Nevertheless, the effective time of these treatments is limited, and the tumors can recur very quickly; hence, the time to re-stenosis of the airway lumen is very short. For tumors outside the airway lumen or at the airway wall, the effectiveness of these methods is even poorer [16–19]. In the present study, CT- and fiber bronchoscopy-guided staging radioactive particle implantation was performed to treat cancerous large airway stenosis. The radioactive particles were implanted permanently under the guidance of fiber bronchoscopy to treat the tumors that caused airway stenosis, which kill the tumor cells continuously [3] and provide continuous high-dose irradiation to irradiate the tumors inside the airway lumen [4, 20], at the airway wall, and around the airway lumen to decrease the size of tumors and enlarge the airway lumen [21]. As the irradiation is continuous, the recurrence of the tumors inside the airway lumen could be delayed and the airway is not obstructed. Therefore, the respiratory functions of the patients are effectively increased, and their quality of life is improved, facilitating eventual further treatments. For the tumors distant from the airway, CT-guided percutaneous ^125^I particle implantation was performed 1 week after the fiber bronchoscopy-guided implantation to ensure that the tumors were irradiated continuously from all directions, hence achieving optimal treatment effects that could not be achieved using external irradiation [5–7].

This treatment method has several advantages: (1) fiber bronchoscopy is minimally invasive, (2) could directionally kill the target cells, (3) only affects the tumors locally, whereas the effects on the normal tissues are limited, (4) effectively solves the issues of high recurrence of the tumors inside the lumen and restores the ventilation functions quickly, (5) kills the tumor cells from all directions, and (6) provides favorable conditions for further treatments, hence improving the quality of life and increasing survival.

## Conclusion

In conclusion, CT- and fiber bronchoscopy-guided staging radioactive particle implantation has definite treatment effectiveness for cancerous large airway stenosis. Indeed, compared with chemotherapy alone, this approach improved the indexes of airway patency, survival, and quality of life. In addition, this method has few minor complications and is easy to perform. Hence, this approach should be widely used in clinical practices.

## Abbreviations

CT: Computed tomography
NSCLC: Non-small cell lung cancer
TPS: Treatment planning system

## References

[1] Tan J, Heriot AG, Mackay J, Van Dyk S, Bressel MA, Fox CD (2013). Prospective single-arm study of intraoperative radiotherapy for locally advanced or recurrent rectal cancer. J Med Imaging Radiat Oncol.

[2] Wu HM, Lu J, Hu WL, Zhang JH, Wang W, Xiao YS (2013). Combination of transrectal 125I seeds implantation brachytherapy and intermittent hormonal therapy for locally advanced prostate cancer. Zhonghua Nan Ke Xue.

[3] van Riet YE, Maaskant AJ, Creemers GJ, van Warmerdam LJ, Jansen FH, van de Velde CJ (2010). Identification of residual breast tumour localization after neo-adjuvant chemotherapy using a radioactive 125 iodine seed. Eur J Surg Oncol.

[4] Shaw Y, Yoneda KY, Chan AL (2012). Cerebral gas embolism from bronchoscopic argon plasma coagulation: a case report. Respiration.

[5] Dauer LT, Thornton C, Miodownik D, Boylan D, Holahan B, King V (2013). Radioactive seed localization with 125I for nonpalpable lesions prior to breast lumpectomy and/or excisional biopsy: methodology, safety, and experience of initial year. Health Phys.

[6] Zhang S, Zheng Y, Yu P, Yu F, Zhang Q, Lv Y (2011). The combined treatment of CT-guided percutaneous 125I seed implantation and chemotherapy for non-small-cell lung cancer. J Cancer Res Clin Oncol.

[7] Matsuoka J, Yashiro M, Doi Y, Fuyuhiro Y, Kato Y, Shinto O (2013). Hypoxia stimulates the EMT of gastric cancer cells through autocrine TGFbeta signaling. PLoS One.

[8] Yoshioka Y (2009). Current status and perspectives of brachytherapy for prostate cancer. Int J Clin Oncol.

[9] Martinez-Monge R, Pagola M, Vivas I, Lopez-Picazo JM (2008). CT-guided permanent brachytherapy for patients with medically inoperable early-stage non-small cell lung cancer (NSCLC). Lung Cancer.

[10] Zalcman G, Bergot E, Lechapt E (2010). Update on nonsmall cell lung cancer. Eur Respir Rev.

[11] Dabrowski A, Ciechanski A, Wallner G, Gorczynski R, Furtak J (2004). Squamous cell oesophageal cancer in patient after surgical treatment of achalasia. Pol Merkur Lekarski.

[12] Cosgrove SE, Ristaino P, Caston-Gaa A, Fellerman DP, Nowakowski EF, Carroll KC (2012). Caveat emptor: the role of suboptimal bronchoscope repair practices by a third-party vendor in a pseudo-outbreak of pseudomonas in bronchoalveolar lavage specimens. Infect Control Hosp Epidemiol.

[13] Szlubowski A, Soja J, Kocon P, Talar P, Czajkowski W, Rudnicka-Sosin L (2012). A comparison of the combined ultrasound of the mediastinum by use of a single ultrasound bronchoscope versus ultrasound bronchoscope plus ultrasound gastroscope in lung cancer staging: a prospective trial. Interact Cardiovasc Thorac Surg.

[14] Qiu M, Xu L, Yang X, Ding X, Hu J, Jiang F (2013). XRCC3 Thr241Met is associated with response to platinum-based chemotherapy but not survival in advanced non-small cell lung cancer. PLoS One.

[15] Mosleh Shirazi MA, Faghihi R, Siavashpour Z, Nedaie HA, Mehdizadeh S, Sina S (2012). Independent evaluation of an in-house brachytherapy treatment planning system using simulation, measurement and calculation methods. J Appl Clin Med Phys.

[16] Portess DI, Bauer G, Hill MA, O'Neill P (2007). Low-dose irradiation of nontransformed cells stimulates the selective removal of precancerous cells via intercellular induction of apoptosis. Cancer Res.

[17] Johnson M, Colonias A, Parda D, Trombetta M, Gayou O, Reitz B (2007). Dosimetric and technical aspects of intraoperative I-125 brachytherapy for stage I non-small cell lung cancer. Phys Med Biol.

[18] Fanta J, Lang O, Vlachova A, Votruba J, Kara J (2006). Lung resection for a non-small cell carcinoma (stage IV) with a permanent intracavitary brachytherapy 125I. Rozhl Chir.

[19] An J, Chervin AS, Nie A, Ducoff HS, Huang Z (2007). Overcoming the radioresistance of prostate cancer cells with a novel Bcl-2 inhibitor. Oncogene.

[20] Trombetta MG, Colonias A, Makishi D, Keenan R, Werts ED, Landreneau R (2008). Tolerance of the aorta using intraoperative iodine-125 interstitial brachytherapy in cancer of the lung. Brachytherapy.

[21] Wu LL, Luo JJ, Yan ZP, Wang JH, Wang XL, Zhang XB (2012). Comparative study of portal vein stent and TACE combined therapy with or without endovascular implantation of iodine-125 seeds strand for treating patients with hepatocellular carcinoma and main portal vein tumor thrombus. Zhonghua Gan Zang Bing Za Zhi.

